# Supplementary material for: Factorial structure of the patient health questionnaire-9, generalized anxiety disorder-7 and berger HIV stigma scale-short form among adolescents living with HIV in Ghana
Source: PLoS One. 2025 Jun 18;20(6):e0326169. doi: 10.1371/journal.pone.0326169 (PMC12176153; doi:10.1371/journal.pone.0326169)
Supplement: S1 File — (DOCX) [file pone.0326169.s001.docx]

**Patient Health Questionnaire**

**Instructions**: Over the last 2 weeks, how often have you been bothered by any of the following problems? (Use “✔” to indicate your answer)

| **Items** | **Not at all** | **Several days** | **More than half the days** | **Nearly every day** |
| --- | --- | --- | --- | --- |
| 1. Little interest or pleasure in doing things |  |  |  |  |
| 1. Feeling down, depressed, or hopeless |  |  |  |  |
| 1. Trouble falling or staying asleep, or sleeping too much |  |  |  |  |
| 1. Feeling tired or having little energy |  |  |  |  |
| 1. Poor appetite or overeating |  |  |  |  |
| 1. Feeling bad about yourself — or that you are a failure or have let yourself or your family down |  |  |  |  |
| 1. Trouble concentrating on things, such as reading the newspaper or watching television |  |  |  |  |
| 1. Moving or speaking so slowly that other people could have noticed? Or the opposite — being so fidgety or restless that you have been moving around a lot more than usual |  |  |  |  |
| 1. Thoughts that you would be better off dead or of hurting yourself in some way |  |  |  |  |

**Scoring:** To score the PHQ-9, add the numbers of all the checked responses under each heading (not at all=0, several days=1, more than half the days=2, and nearly every day=3). High scores represent more depressive symptoms.

**Generalised Anxiety Disorder (GAD-7)**

**Instructions:** The following questions ask about how you have been feeling over the past two weeks. Please carefully read each statement and indicate how often you have been bothered using ‘√’ to indicate your answer.

| Items | Not at all sure | Several days | **More than half the days** | Nearly every day |
| --- | --- | --- | --- | --- |
| Over the last two weeks, how often have you been bothered about the following problems |  |  |  |  |
| 1. Feeling nervous, anxious, or on edge |  |  |  |  |
| 1. Not being able to stop or control worrying |  |  |  |  |
| 1. Worrying too much about different things |  |  |  |  |
| 1. Trouble relaxing |  |  |  |  |
| 1. Being so restless that it's hard to sit still |  |  |  |  |
| 1. Becoming easily annoyed or Irritable |  |  |  |  |
| 1. Feeling afraid as if something awful might happen |  |  |  |  |

Scoring: To score GAD-7, add the numbers of all the checked responses under each heading (not at all=0, several days=1, more than half the days=2, and nearly every day=3). High scores represent more anxiety symptoms.

**Berger Stigma Scale**

**Instructions:** Below are several statements related to how people with HIV may feel or experience interactions with others. Please read each statement carefully and indicate how much you agree or disagree with each one by ticking (√) in the boxes.

| **Items** | Strongly disagree | Disagree | Neutral | Agree | Strongly agree |
| --- | --- | --- | --- | --- | --- |
| 1. I have been hurt by how people reacted to learning I have HIV. |  |  |  |  |  |
| 1. I have stopped socializing with some people because of their reactions to my having HIV |  |  |  |  |  |
| 1. I have lost friends by telling them I have HIV |  |  |  |  |  |
| 1. I am very careful who I tell that I have HIV. |  |  |  |  |  |
| 1. I worry that people who know I have HIV will tell others. |  |  |  |  |  |
| 1. I feel that I am not as good a person as others because I have HIV. |  |  |  |  |  |
| 1. Having HIV makes me feel unclean |  |  |  |  |  |
| 1. Having HIV makes me feel that I’m a bad person |  |  |  |  |  |
| 1. Most people think that a person with HIV is disgusting |  |  |  |  |  |
| 1. Most people with HIV are rejected when others find out |  |  |  |  |  |

Scoring: To score the Berger Stigma Scale, add the numbers of all the checked responses under each heading (Strongly disagree=1, Disagree=2, Neutral=3, Agree=5 and Strongly agree=3). High scores represent more stigma experience.
