# Supplementary material for: Factorial structure of the patient health questionnaire-9, generalized anxiety disorder-7 and berger HIV stigma scale-short form among adolescents living with HIV in Ghana
Source: PLoS One. 2025 Jun 18;20(6):e0326169. doi: 10.1371/journal.pone.0326169 (PMC12176153; doi:10.1371/journal.pone.0326169)
Supplement: Attachment — Submitted filename: Response to Reviewer Comments.docx [file pone.0326169.s003.docx]

Factorial structure of the Patient Health Questionnaire -9(PHQ-9) Generalized Anxiety Disorder-7(GAD-7) and Berger HIV Stigma Scale-Short Form (HSS-SF) among Adolescents living with HIV in Ghana

**PONE-D-25-17680**

We have noted our responses to the reviewer comments, and these have been highlighted in yellow ink for quick reference.

**Reviewer #1:**

1. It is recommended that the discussion section further address cultural differences in the experience of stigma and mental health.

Authors response: We are grateful for this feedback. We have incoporated this into the revised manuscript (page 20, line 391)

“Furthermore, the two-factor structure of PHQ-9 identified in our Ghanaian sample may reflect culture-specific expressions of depression among ALHIV in West African contexts. In collectivistic cultures like Ghana, mental health symptoms often manifest through somatic complaints rather than direct expression of emotional distress, which may explain the distinct cognitive/affective and somatic domains found in our analysis (Anum et al., 2019). This finding aligns with research indicating that cultural factors significantly influence how mental health symptoms are experienced and reported across different populations (Hwang et al., 2008). The sociocultural context of Ghana, characterized by strong family ties, religious beliefs, and communal living arrangements, creates a unique environment in which stigma and mental health challenges manifest. Ghanaian adolescents living with HIV often experience stigma shaped by cultural beliefs about illness causation, moral judgments, and fears of contagion that may differ from those in Western settings. The measurement of these experiences requires culturally sensitive approaches that acknowledge these distinctions. This notwithstanding, the PHQ-9 and GAD-7 have demonstrated good factorial structure, internal consistency and correlations among ALHIV in Ghana.”

2. It is also suggested that measurement invariance across subgroups (e.g., age or gender) be examined to enhance the generalizability of the findings.

Authors response: Thank you very much for the suggestion. Indeed, it was part of our original plan to conduct gender invariance analysis. Unfortunately, the sample size was not only small but also unevenly distributed. The boys were only 28. We have acknowledged this as potential limitation towards gender specific screening, as follows:

“The uneven distribution of the participants based on gender (28 males versus 77 females) also restricted gender invariance testing, thereby limiting discussions on the utility of the measures for gender-specific screening.” (page 22, line 448).

3. Concurrent validity with clinical measures is also recommended for future studies.
Authors response: Thank you very much. We have noted this and will incorporate same into our future endeavors.

4. Overall, this manuscript represents an excellent example of instrument validation in a distinct cultural context and could serve as a valuable regional reference.

Authors response: We are very grateful for the positive feedback.

**Reviewer #2**

**Comment 1:** The paper is a quantitative study entitled ‘Factorial structure of the Patient Health Questionnaire -9(PHQ-9) Generalized Anxiety Disorder-7(GAD-7) and Berger HIV Stigma Scale-Short Form (HSS-SF) among Adolescents living with HIV in Ghana ‘This title is a bit too long. It would be better if the authors avoided abbreviations in the title but have them in the main text.

Authors response: Thank you very much. We agree with you to remove the abbreviations from the title. The manuscript has been revised accordingly, as follows:

“Factorial Structure of the Patient Health Questionnaire-9, Generalized Anxiety Disorder-7 and Berger HIV Stigma Scale-Short Form among Adolescents Living with HIV in Ghana.”

Abstract
**Comment 2:** The abstract is well detailed and addresses the main research question. The study addressed one main objective namely, the factor structure, internal consistency, and correlations of the PHQ-9, GAD-7, and HSS-SF among ALHIV in Ghana. This is well captured in the abstract.

Authors response: Thank you for the positive feedback.

Introduction
**Comment 3:** The introduction and background information are nicely detailed and has clearly identified the gap in the usage of the 3 tools.

Authors response: Thank you for the positive feedback.

Materials and Methods
**Comment 4**: The study design, setting, exclusion and inclusion criteria are well explained but the sampling procedure is unclear. In line 174-175, the authors should state, out of the total percentage of the required population what percentage was 63 for Atua, 16 for Akuse and 26 for Asesewa. Being a quantitative study it is important to explain how the sample size was arrived at. The scope of the study is well provided and the presentation is consistent throughout the manuscript.

Authors response: We have revised the manuscript to provide additional information on sample size as follows:

In Atua, of the total of 120 adolescents living with HIV within the required age range, only 75 fully satisfied the inclusion criteria (thus awareness of HIV status for at least 6 months). Out of this only 63 provided informed consent and assent and were available for data collection, representing 60% of the final sample. In Akuse**,** of the total of 55 ALHIV within the required age range, only 20 had an awareness of their HIV status for at least 6 months. Out of these,16 consented and participated in the data collection, representing 15% of the final sample. In Asesewa**,** of the total of 72 ALHIV within the required age range, only 30 satisfied the criteria: awareness of HIV status for at least 6 months. Out of these, 26 provided informed consent and assent and participated in the study, representing 25% of the final sample. The final sample size of 105 participants (63+16+26) was therefore determined by the number of eligible adolescents who both met the inclusion criteria and provided informed consent across all three study locations. This represents an overall response rate of 84% (105/125) among those who met the inclusion criteria. (page 8, line 175).

**Comment 5**: Data collection procedure
This section is detailed and well written.

Authors response: Thank you for the positive feedback.

Data analysis and Results
**Comment 6:** The findings that the study produces are very critical but have no implications, and they only need one or two sentences to bring out the implication of each finding so that the study remains in context.

Authors response: We have incorporated clinical implications of the study findings as follows:

**Clinical implications of the study**

“In Ghana and several other African countries, there are growing calls and concerted efforts to integrate mental health into primary healthcare for all categories of patients, including ALHIV. The availability of validated and psychometrically sound measures will contribute significantly towards the realization of integrated healthcare, by facilitating screening for mental health issues for early intervention. The study contributed to this growing agenda by providing preliminary evidence supporting the factor structure and reliability of the PHQ-9, GAD-7 and Berger HSS-SF among ALHIV. To proceed in this regard, healthcare professionals can be trained to administer the PHQ-9, GAD-7 and Berger HSS-SF as part of the routine healthcare services to ALHIV to support evidence-based decision making such as recommendations for detailed assessment and intervention programming to improve their mental health and wellbeing. Early screening and detection would support early intervention programming to address subliminal mental health problems from developing into full-blown, diagnosable mental health disorders among ALHIV.” (page 22, line 428).

**Comment 7:** The manuscript is well written with key subtopics availed. The authors however need to be more analytical in data presentation. The results are presented and not interpreted at all. Making it difficult to make exact meaning. It is always good practice to interpret all findings, this makes it easy to come up with the implications of the findings. The manuscript would be more useful to a broader readership if the authors moved from just providing results to interpreting them and giving the implication as well.

Authors response: Thank you very much for this response. We have interpreted key findings in the result section. These have been marked in yellow ink.

**Comment 8:** I tend to think that the authors oversimplified the findings thus inhibiting the deeper message that the subject under study could provide.

Authors response: Thank you very much for the observation. We are cautious not to bite than we can chew. This is among the few studies and the first in Ghana and perhaps West African region to have examined the factorial structure of the study measures among ALHIV. As a result, we can only focus on the main focus of the paper which is the factor structure of the measures. We did not explore other issues that would require some detailed and connected discussions. We are grateful.

Discussion
**Comment 9**: On the PHQ-9 the authors propose that it would be beneficial to explore the affective/ cognitive and somatic domains separately (Line 347). How about separating cognitive and affective domains or why do the authors think that cognitive and affective should be studied together in the case of Ghana. Generally, the discussion is good but as a wrap up what was the most common finding of this study and what research gap does it fill? Or what new information came from this study? The discussion section should be improved by linking the study findings to earlier works on the subject area and explaining how the current study complements previous ones.

Authors response: Thank you very much for the observation. Regarding studying cognitive/affective and somatic domains, it appears that we did not express ourselves well, leading to confusion in understanding our position. We wrote that

“Therefore, our study proposes that for ALHIV, it would be beneficial to explore their affective/cognitive and somatic domains separately to obtain nuanced information about their mental health and wellbeing to inform both research and clinical practice.” To ensure clarity, we have offered additional discussion as follows “Stated alternatively, the PHQ-9 should be seen as assessing different categories of depressive symptoms, namely cognitive/affective symptoms and somatic symptoms. This will help to give a better representation and distribution of the depressive tendencies among ALHIV. For a nuanced understanding of depressive symptoms, the study did not support previous endorsement of a unidimensional factor structure of the PHQ-9, although total depressive score can be obtained by summing the scores for both cognitive/affective symptoms and somatic domains.” (page 19, line 367)

As you can see, our intention is not to suggest that cognitive/affective and somatic domains should be explored separately….We have review the manuscript, ensuring that the key message of the study stands out.

Limitations
**Comment 10**: What about the sociocultural influences on the responses of the adolescents? Don’t you think this is a limitation?

Authors response: Thank you for bringing this to our attention. We have incorporated this into the revised manuscript.

“The reliance on self-report measures and face-to-face interviews may have introduced social desirability bias, particularly given the sensitive nature of questions about mental health and stigma experiences. Indeed, the Ghanaian society and cultures stigmatize HIV, culminating into discriminatory practices and other negative behaviors. These experiences could potentially bias their responses. Notwithstanding the limitations above, the preliminary findings reported on the factor structure of the PHQ-9 and GAD-9 among ALHIV in Ghana are consistent with previous studies (Stanyte et al., 2023), thereby giving some credence to the study.” (Page 23, line 456).

Conclusion
**Comment 11**: The conclusion is good except line 401-402-what did HSS-SF demonstrate? It would be nice to state the findings before indicating that they are not sufficient and acceptable.

Authors response: Thank you very much for this response. The revised manuscript reads:

“The Berger HSS-SF was adequately represented by a three-factor structure instead of the original four factors. The deletion of the Public Disclosure subscale improved the factor structure of the Berger HSS-SF. The finding raises questions about the utility of the Public Disclosure in ALHIV discourse, necessitating the call for future research.” (page 23-24, line 472).

References
